# The Hydrophobic Core of Twin-Arginine Signal Sequences Orchestrates Specific Binding to Tat-Pathway Related Chaperones

**DOI:** 10.1371/journal.pone.0034159

**Published:** 2012-03-30

**Authors:** Anitha Shanmugham, Adil Bakayan, Petra Völler, Joost Grosveld, Holger Lill, Yves J. M. Bollen

**Affiliations:** Department of Molecular Cell Biology, VU University Amsterdam, Amsterdam, The Netherlands; MRC National Institute for Medical Research, United Kingdom

## Abstract

Redox enzyme maturation proteins (REMPs) bind pre-proteins destined for translocation across the bacterial cytoplasmic membrane via the twin-arginine translocation system and enable the enzymatic incorporation of complex cofactors. Most REMPs recognize one specific pre-protein. The recognition site usually resides in the N-terminal signal sequence. REMP binding protects signal peptides against degradation by proteases. REMPs are also believed to prevent binding of immature pre-proteins to the translocon. The main aim of this work was to better understand the interaction between REMPs and substrate signal sequences. Two REMPs were investigated: DmsD (specific for dimethylsulfoxide reductase, DmsA) and TorD (specific for trimethylamine N-oxide reductase, TorA). Green fluorescent protein (GFP) was genetically fused behind the signal sequences of TorA and DmsA. This ensures native behavior of the respective signal sequence and excludes any effects mediated by the mature domain of the pre-protein. Surface plasmon resonance analysis revealed that these chimeric pre-proteins specifically bind to the cognate REMP. Furthermore, the region of the signal sequence that is responsible for specific binding to the corresponding REMP was identified by creating region-swapped chimeric signal sequences, containing parts of both the TorA and DmsA signal sequences. Surprisingly, specificity is not encoded in the highly variable positively charged N-terminal region of the signal sequence, but in the more similar hydrophobic C-terminal parts. Interestingly, binding of DmsD to its model substrate reduced membrane binding of the pre-protein. This property could link REMP-signal peptide binding to its reported proofreading function.

## Introduction

Twin-arginine transport (Tat) systems are present in plant chloroplasts and many prokaryotes. The *Escherichia coli* Tat system was first identified as a transporter of extracellular redox enzymes that require cofactor insertion and assembly in the cytoplasm prior to transport [1]. Later, a broader range of proteins was added to the list of *E. coli* Tat substrates. The *E. coli* Tat translocon consists of the proteins TatA/TatE, TatB, and TatC [2]. There is currently no explicit evidence for additional proteins other than TatA/EBC essential for transport. However, there are instances where cytoplasmic chaperones play a role along the Tat pathway, presumably assisting the folding of substrates (for a review see [3]). Recent evidence demonstrates that *E. coli* DnaK is directly involved in the Tat pathway [4], [5] but the involvement of other general *E. coli* chaperones, e.g. GroEL and SlyD, remains obscure owing to their largely overlapping functions.

In addition to general chaperones, a class of substrate-specific redox enzyme maturation proteins (REMPs) has been identified (reviewed in [6]). REMPs assist in the intracellular incorporation of complex cofactors into extracellular redox enzymes prior to Tat-dependent translocation. Usually, each REMP recognizes a single specific substrate protein. Several cognate REMP-substrate pairs have been identified. The two best-characterized REMPs are DmsD and TorD. TorD binds to trimethylamine N-oxide reductase (TorA) and assists in the incorporation of the molybdo-bis(molybdopterin guanine dinucleotide) cofactor. DmsD binds to dimethylsulfoxide reductase (DmsA) as well as to two homologous proteins YnfE and YnfF, and keeps these proteins in a conformation that allows incorporation of the same molybdo-bis(molybdopterin guanine dinucleotide) cofactor by other proteins.

Similar to substrates of the ubiquitous general secretory (Sec) pathway, Tat substrates bear a transport-essential N-terminal signal sequence that consists of a positively charged N-region, a hydrophobic H-region and a short polar C-region. However Tat signal sequences bear several additional features [7]: the N-region is usually longer than is the case for Sec substrates; a consensus S/TRRxFLK motif containing the highly conserved name-giving twin-arginine pair is found at the boundary between N- and H-regions; the H-region is less hydrophobic than is the case for Sec signal peptides; and finally the C-region usually contains at least one positively charged amino acid residue, sometimes referred to as the “Sec-avoidance motif” [8].

Tat substrate signal sequences are involved in at least three important steps along the translocation path. First, the signal peptide is involved in the recognition of Tat substrates by REMPs. For example DmsD has been shown to directly bind to the signal peptide of DmsA [9]. Recently, it was shown that many (but not all) REMPs bind to the signal peptides of the binding partner [10]. Second, signal peptides bind to phospholipid bilayers and biological membranes, possible corresponding to the step that directly precedes TatC binding [11], [12]. Third, signal peptides are recognized and bound by TatC, which is presumably a trigger for translocation [13].

REMPs are considered bifunctional chaperones, with roles in both the maturation and transport of substrates. The function in maturation is to keep the substrate in a conformation in which it can bind its redox cofactor [14], [15]. The role in transport is less clear. Initially, it was thought that DmsD directs DmsA to the Tat translocon [16]. However, other data showed that DmsD does not function as a targeting factor [17]. REMPs were also shown to protect the signal peptide from degradation by peptidases [18], [19]. It has been proposed that REMPs prevent the premature interaction of immature substrates with the Tat system which only transports fully folded proteins [20], [21]. Unfolded polypeptides can bind to the TatBC receptor complex, but cannot be transported [22]. Such binding of an unfolded polypeptide to TatBC blocks the Tat system and leads to severe growth defects [22]. REMPs are supposed to prevent such premature interactions, a process sometimes referred to as ‘proofreading’ [23]. However, there is little experimental evidence to directly support this hypothesis.

An open question is: what is the function of the two arginine residues (RR) in the signal sequence? They are known to be essential for translocation. Even the most conservative mutation to two lysine residues blocks translocation entirely [24], [25]. For the Tat substrate SufI it has been shown that the two arginines are essential for binding to TatC, but not for binding to the membrane [12]. Whether the two arginines are important for binding to REMPs is not clear. On one hand, Hatzixanthis *et al.* have analyzed the binding of the TorA signal peptide to TorD by isothermal titration calorimetry (ITC). They found that the twin-arginine motif is probably not required for TorD binding [26]. On the other hand, Stevens *et al.* have analyzed the interaction between DmsD and the DmsA signal peptide using docking and molecular dynamics simulations, and concluded that the twin-arginine motif probably interacts with conserved regions on the surface of DmsD [27]. In summary, the interactions between REMPs and their substrates, and the actual function of this interaction in the twin-arginine transport pathway are still not well understood.

The work presented here aims to provide more insight into the nature of the interactions between Tat signal sequences and REMPs, and to better understand the function of REMP binding in the Tat pathway. In particular, we focus on two questions: (*i*) how do REMPs discriminate between their cognate substrate(s) and other Tat substrates, which often have many similar features; and (*ii*) how does REMP binding affect signal-peptide binding to the membrane relating to the next step in the Tat translocation pathway. Chimeric substrates were created based on green fluorescent protein (GFP) fused to different signal sequences. This approach has three important features: (*i*) GFP reports on the folding state of the pre-protein: it is only fluorescent when fully folded [28]; (*ii*) the same mature domain is used in all experiments, which ensures that observed differences in REMP binding are due to the signal sequence, and not to the mature domain; and (*iii*) such chimeric pre-proteins are functional Tat substrates, since their expression in *E. coli* results in periplasmic accumulation of GFP [11], [29]. The signal peptides thus behave naturally when fused to GFP. Using surface plasmon resonance (SPR) it is shown that the signal sequences of TorA and DmsA target the cargo GFP-fusion protein to the corresponding REMP. The targeting information resides in the combined H- and C-regions, which for TorA and DmsA are substantially hydrophobic and rather similar in overall composition. The N-region, which includes the twin-arginine motif but otherwise differs considerably between TorA and DmsA, is not involved in providing specificity to the interaction with the REMP. Interestingly, the presence of DmsD reduces membrane binding of ssDmsA-GFP, which might be important in preventing the premature interaction of the pre-protein with the Tat system.

## Materials and Methods

### Plasmids and bacterial strains

Wild-type signal sequences were amplified from the *torA* and *dmsA* genes, which had previously been amplified from the *E. coli* genome and cloned into vector pBSK. Hybrid signal sequences combining N-, H-, and C-regions from *torA* and *dmsA* were created entirely by oligonucleotide synthesis and cloned into pUC57 (Eurogentec, Maastricht, The Netherlands). Subsequently, signal sequences were amplified from pBSK or pUC57 by PCR, and at the same time given a 3′-extension that corresponds to the first 15 basepairs of the GFP insert. In a second PCR reaction the signal sequences were fused to the GFP insert and cloned into pTYB11 (New England Biolabs, Ipswich, MA, USA) such that a single fusion protein is encoded consisting of an intein domain, a signal sequence and GFP. Mutations in the twin-arginine regions of the signal sequences were subsequently created by site-directed mutagenesis. pGFP6A, encoding Strep-GFP, was made as follows. First, the internal NdeI restriction site in the GFP gene was removed by site-directed mutagenesis. Subsequently, the mutant GFP gene was amplified by pcr and cloned into the NdeI/BamHI restriction sites of pET3a (New England Biolabs), resulting in pGFP4. Finally, pGFP4 was restricted by NdeI and ligated with two oligonucleotides such that a StrepII-tag is encoded upstream of the GFP-gene. An overview of the expression plasmids used is given in Table 1.

**Table 1:** List of plasmids used in the study.

*Plasmid*	*Reference*	*Protein of interest*
pTYB11-TorA-GFP	[11]	ssTorA-GFP with self cleavable intein tag
pTYB11-TorA(KR)-GFP	This work	ssTorA(KR)-GFP with self cleavable intein tag
pTYB11-TorA(RK)-GFP	This work	ssTorA(RK)-GFP with self cleavable intein tag
pTYB11-TorA(KK)-GFP	This work	ssTorA(KK)-GFP with self cleavable intein tag
pTYB11-TorA-KKK-GFP	This work	ssTorA(KKK)-GFP with self cleavable intein tag
pTYB11-DmsA-GFP	[11]	ssDmsA-GFP with self cleavable intein tag
pTYB11-DmsA(KK)-GFP	This work	ssDmsA(KK)-GFP with self cleavable intein tag
pTYB11-TTD-GFP	This work	ssTorA-GFP with self cleavable intein tag, with the C-region of ssDmsA
pTYB11-TDT-GFP	This work	ssTorA-GFP with self cleavable intein tag, with the H-region of ssDmsA
pTYB11-DTT-GFP	This work	ssTorA-GFP with self cleavable intein tag, with the N-region of ssDmsA
pTYB11-DDT-GFP	This work	ssDmsA-GFP with self cleavable intein tag, with the C-region of ssTorA
pTYB11-DTD-GFP	This work	ssDmsA-GFP with self cleavable intein tag, with the H-region of ssTorA
pTYB11-TDD-GFP	This work	ssDmsA-GFP with self cleavable intein tag, with the N-region of ssTorA
pET16b-TorD	This work	TorD with N-terminal His-tag
pET16b-DmsD	This work	DmsD with N-terminal His-tag
pGFP6A	[38]	Strep-GFP

To express TorD and DmsD, *E. coli torD* and *dmsD* genes were amplified from the genome and cloned into vector pET16b (Invitrogen), which encodes an N-terminal His-tag. *E. coli* strains used in this study were MC4100 (*F^−^ araD139 Δ(argF-lac)U169 rpsL150 relA1 deoC1 rbsR fthD5301 fruA25 λ^−^*), DADE (MC4100 *ΔtatABCD, ΔtatE*, [30]), and BL21(DE3)*.

### Protein expression and purification

For large-scale preparation, the intein-fused signal GFPs were expressed in *E. coli* BL21(DE3)* cells and column purified as described in previous work [11]. In short, the fusion proteins were bound to a chitin affinity column (New England Biolabs) via the chitin-binding domain that is engineered into the intein domain. Subsequently, self-cleavage of the intein domain is induced by reduction on the column, and GFP is released with an intact and unmodified signal sequence at its N-terminus. StrepII-tagged GFP was expressed from pGFP6A in BL21(DE3)* cells and purified using Strep-Tactin Superflow resin (IBA, Göttingen, Germany) as recommended by the manufacturer. His-tagged TorD and DmsD were also expressed in *E. coli* BL21(DE3)* cells and column purified as recommended in the manual, “The Qiaexpressionist” (Qiagen).

### SPR measurements

Surface plasmon resonance measurements were performed on Biacore 2000 or Biacore T100 systems (Biacore AB, Uppsala, Sweden) at 25°C and a flow rate of 50 µl/min, unless indicated otherwise. All solutions were freshly prepared, degassed, and filtered through membranes with 0.22 µm pores. Nitriloacetic acid (NTA) chips providing affinity for Ni-ions were used to study interaction between pre-proteins and REMPs. The running buffer was 10 mM 4-(2-hydroxyethyl)-1-piperazineethanesulfonic acid (HEPES), 150 mM NaCl, 50 µM ethylenediaminetetraacetic acid (EDTA), and 0.005% P20 detergent at pH 7.4. In each cycle, 50 µl of 0.5 mM NiCl_2_ in running buffer was injected to activate the surface, followed by injection of His-tagged REMP in running buffer, except for the reference surface, to which no protein was bound. For qualitative binding studies, REMPs were immobilized to a readout of 1000 response units (RU) in order to maximize the sensitivity of the experiment. For quantitative experiments in which dissociation constants were determined, a lower level of immobilization (500 RU) was employed, to minimize non-specific binding of the pre-proteins to the reference surface. The concentration of pre-proteins was varied between 10 and 6400 nM. After each interaction measurement, the SPR chip was regenerated with 250 mM EDTA.

L1 sensor chips (Biacore AB) on which lipophilic groups are covalently attached to the surface via a dextran matrix were used to study protein interactions with membranes. Small unilamellar vesicles (SUVs) were generated from *E. coli* membranes or from lipids mimicking the *E. coli* inner membrane composition as described in our previous work [11]. SUVs were spread and immobilized on the sensor chip [11]. After completion of an experiment, the surface of the chip was regenerated by washing with 40 mM *N*-octyl -D-glucopyranoside.

### Sequence alignment

The signal sequences of TorA (residues 1–42), DmsA (residues 1–45), YnfE (residues 1–43) and YnfF (residues 1–49) were aligned using the online tool ClustalW (version 1.83, http://www.ch.embnet.org/cgi-bin/clustalw_parser) with default parameters: open gap penalty 10, extending gap penalty 0.05, an end gap penalty 10, and a separation gap penalty 0.05.

## Results

### Specific interaction of REMPs with chimeric Tat pre-proteins

**Figure 1:**
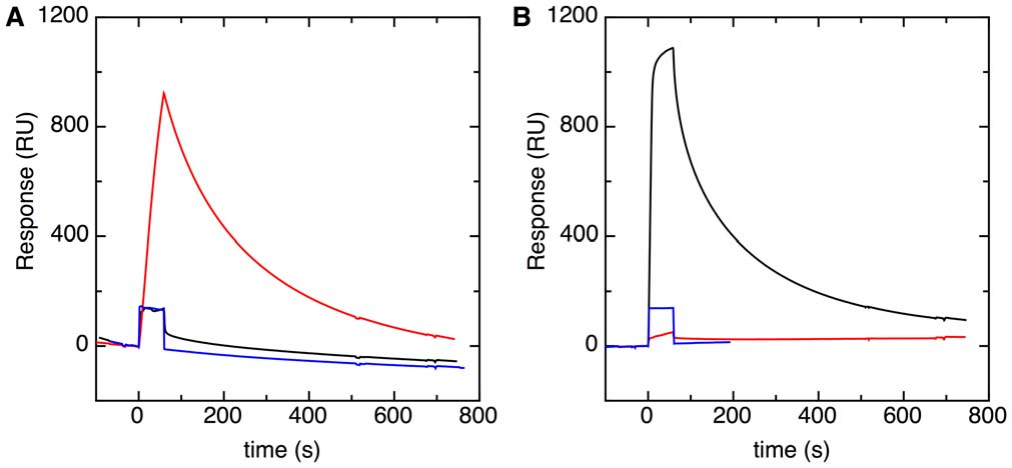
The signal sequence ensures specific REMP binding. Surface plasmon resonance sensorgrams for the injection of various proteins over immobilized TorD (**A**) and DmsD (**B**) are shown. The model pre-proteins ssDmsA-GFP (black lines), ssTorA-GFP (red lines) and signal sequence-free GFP (blue lines) were injected for 60 seconds at a concentration of 200 nM, a flow rate of 50 µl/min, and a temperature of 25°C.

Binding of the Tat signal peptides to REMPs was investigated by SPR. His-tagged DmsD and TorD were immobilized in separate lanes on the surface of a Ni-NTA chip. The signal sequence of DmsA fused to GFP (ssDmsA-GFP) and the signal sequence of TorA fused to GFP (ssTorA-GFP) were separately injected over this surface for a time period of 60 s. The observed SPR responses (Figure 1) imply that ssDmsA-GFP binds to the DmsD covered surface, but not to the TorD surface. Similarly ssTorA-GFP shows specific binding to TorD. GFP by itself did not bind to either TorD or DmsD (Figure 1); the step-like SPR responses observed are a direct result of the higher refractive index of the protein solution compared to the protein-free buffer. The specific interactions between the REMPs and their cognate GFP-fusion pre-proteins thus are apparently governed by the respective Tat signal peptide.

### Affinity constants for the interaction between REMPs and pre-proteins

**Figure 2:**
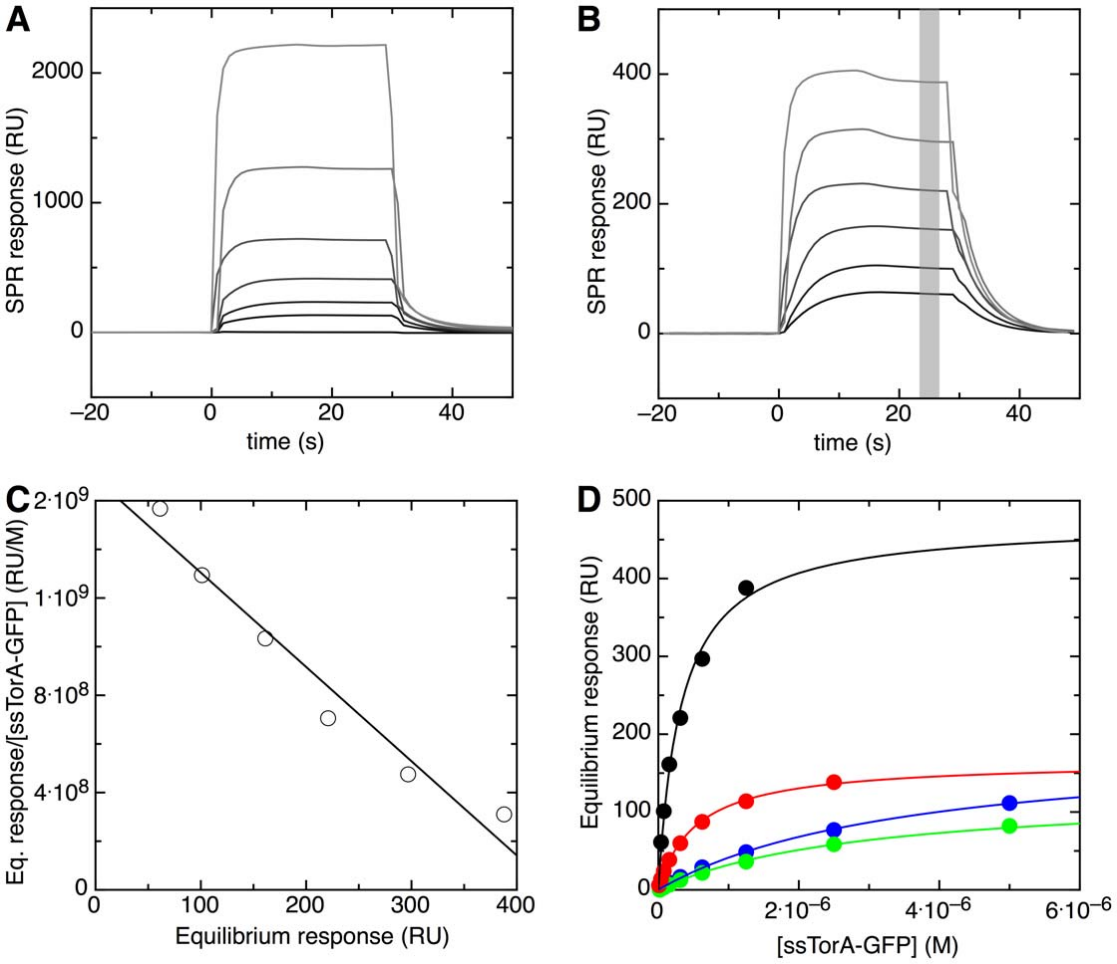
Determination of the dissociation constant for ssTorA-GFP binding to immobilized TorD. (**A**) Raw SPR sensorgrams and (**B**) referenced sensorgrams of ssTorA-GFP binding to immobilized TorD at the following pre-protein concentrations (shown from dark to light grey): 39 nM, 78 nM, 156 nM, 313 nM, 625 nM, 1250 nM. The sensorgram for a buffer injection is shown in black. (**C**) Equilibrium SPR intensity for binding of ssTorA-GFP to TorD at various concentrations in a Scatchard plot (open symbols, see main text for details). The intensity values used are the average SPR response derived from the shaded area (22–28 s) in panel A. The data are fitted to a straight line (black). (**D**) The SPR intensity at equilibrium is plotted as a function of pre-protein concentration, for binding to immobilized TorD. The pre-proteins used are ssTorA-GFP (shown in black), ssTorA(RK)-GFP (shown in blue), ssTorA(KR)-GFP (shown in green) and ssTorA(KK)-GFP (shown in red). The best fit of the Langmuir binding isotherm (Equation 1) to the data is shown as a line in the corresponding color.

The affinity of TorD for the signal sequence of TorA was quantified by recording SPR response curves at different pre-protein concentrations (Figure 2A). A low flow rate of 20 µl/min was used to increase the contact time and hence allow the binding reaction to equilibrate.. To test whether non-specific binding of ssTorA-GFP directly to the Ni-NTA surface contributes to the SPR curves, the same pre-protein solutions were also injected over a reference surface to which no TorD was immobilized. Since binding of ssTorA-GFP to this reference surface was indeed observed, the raw SPR curves were corrected for non-specific binding by subtracting the reference curves. The resulting curves are shown in Figure 2B. A small drop is observed during the injection phase of the SPR response curves at the highest pre-protein concentrations, approximately 15 seconds after injection. This drop is hardly present in the raw data (Figure 2A), and thus results from the correction procedure. This is probably caused by the following. An unliganded Ni-NTA surface has more Ni^2+^ ions available for unspecific binding to ssTorA-GFP than a surface to which TorD is bound. As a result, subtraction of the control curve results in an over-correction for unspecific binding, which leads to the observed drop in the SPR intensity. Fortunately, the effect is small and mainly seen at the highest pre-protein concentrations. We therefore continued to quantify the corrected SPR curves. The SPR intensity at equilibrium (*I_eq_*) was determined as the average of the signal intensity over the shaded region in Figure 2A at each pre-protein concentration. The data are represented in a Scatchard plot, in which *I_eq_* divided by the concentration of pre-protein is plotted against *I_eq_* (Figure 2C). A linear function describes the data well, which is a strong indication of equimolar binding. *I_eq_* was then plotted against the concentration of pre-protein (Figure 2D), and fitted using a Langmuir model for 1∶1 binding
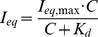
(1)
*I_eq,max_* is the equilibrium SPR intensity at a saturating pre-protein concentration, *C* is the concentration of pre-protein and *K_d_* is the dissociation constant of the pre-protein - REMP complex. The resulting *K_d_* for TorD:ssTorA-GFP is 0.33±0.04 µM (Table 2).

**Table 2:** Dissociation constants for TorA signal peptides binding to TorD.

Signal sequence	*K_d_* (µM)^1^	Method	Construct^2^	Reference
TorA	0.33±0.04	SPR	ss-GFP	this work
TorA(KR)	2.9±0.1	SPR	ss-GFP	this work
TorA(RK)	3.6±0.2	SPR	ss-GFP	this work
TorA(KK)	0.54±0.04	SPR	ss-GFP	this work
TorA(KKK)	4.9±0.1	SPR	ss-GFP	this work
TorA	1.7	ITC	peptide S10-R26	[26]
TorA	0.059	ITC	MBP-ss	[32]
TorA	3.8	SPR	ss-SBP	[19]

1 Dissociation constants from this work are obtained from SPR data by fitting the Langmuir equation (Equation 1) to the equilibrium SPR response as a function of pre-protein concentration. Errors are standard fitting errors.

2 ss, signal sequence; GFP, green fluorescent protein; MBP, maltose binding protein; SBP, streptavidin binding peptide.

The same approach was followed for the binding of ssDmsA-GFP to DmsD. However, in this case the Scatchard plot was non-linear, which indicated that the binding of ssDmsA-GFP to DmsD did not follow a 1∶1 model. Hence the Langmuir equation does not apply, and the *K_d_*-value could not be reliably determined.

### The role of the twin-arginine motif in pre-protein binding to REMPs

To determine whether the double arginine motif in the TorA signal peptide has any role in pre-protein binding to TorD, either one or both of the twin-arginine motif residues (RR) was mutated to lysine, resulting in ssTorA(RK)-GFP, ssTorA(KR)-GFP, and ssTorA(KK)-GFP. These mutations are known to affect translocation: ssTorA(RK)-GFP and ssTorA(KR)-GFP show strongly reduced translocation efficiencies, whereas ssTorA(KK)-GFP is not transported at all [24], [25]. The mutant pre-proteins were purified and their affinity for TorD was measured by SPR (Figure 2 D) and quantified using Equation 1. All three mutant pre-proteins bind approximately ten-fold more weakly compared to the wild-type ssTorA-GFP (Table 2).

**Figure 3:**
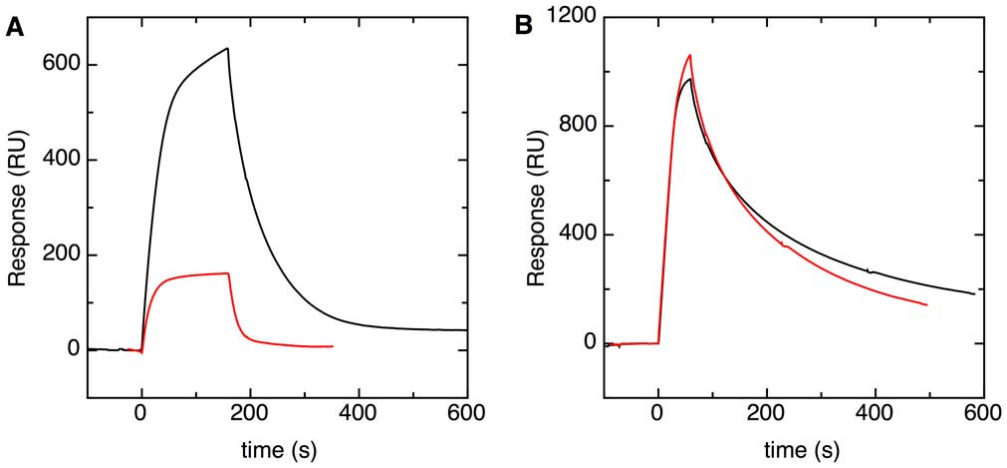
Twin-arginines are not essential for REMP binding. (**A**) SPR response curves obtained by injecting solutions containing 100 nM ssTorA-GFP (black line) or 100 nM ssTorA(KKK)-GFP (red line) over immobilized TorD. (**B**) Response curves obtained by injecting solutions of 100 nM ssDmsA-GFP (black line) or 100 nM ssDmsA(KK)-GFP (red line) over immobilized DmsD.

In the signal sequence of TorA, the residue following the two conserved arginine residues happens to be an arginine as well. In order to rule out that the third arginine takes over any role of the two conserved ones in the binding to TorD, this residue was also mutated to a lysine, resulting in ssTorA(KKK)-GFP. The dissociation constant of 4.9 µM found for ssTorA(KKK)-GFP binding to TorD is similar to that obtained for ssTorA(KR)-GFP and ssTorA(RK)-GFP, and approximately ten-fold higher than for the wild-type signal sequence (Figure 3A, Table 2). Moreover, a more drastic mutation of signal sequence was made in which the three arginine residues were changed to the tripeptide glycine-histidine-proline (ssTorA(GHP)-GFP). This pre-protein was expressed, purified and tested for binding to TorD. However, no binding could be detected (data not shown).

The two arginines in the signal peptide of DmsA were also mutated to lysine. The SPR curve for a 100 nM solution of the pre-protein ssDmsA(KK)-GFP binding to DmsD was qualitatively very similar to that for wild-type ssDmsA-GFP at the same concentration (Figure 3B). Moreover the SPR intensities at equilibrium were very similar for both pre-proteins (not shown). For ssDmsA(KK)-GFP, binding of the mutant signal sequence to DmsD could not be readily quantified because the interaction does not follow the 1∶1 binding model. Nevertheless, it can be concluded that conservative mutation of the RR-motif to lysine(s) has very little effect on ssDmsA-GFP binding to DmsD.

In conclusion, the conserved arginine pair in the signal peptides of the Tat substrates TorA and DmsA does not absolutely determine binding of the peptides to the cognate REMP. Conservative mutations to lysine residues are tolerated. However, for ssTorA binding to TorD more drastic mutations in which the positively charged residues are removed result in loss of binding.

### Specific binding depends on H- and C-regions

**Figure 4:**
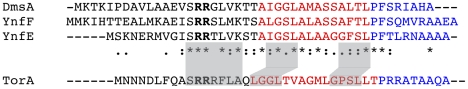
Alignment of DmsA and TorA signal sequences. The signal sequences of the DmsD substrates DmsA, YnfF and YnfE were aligned using ClustalW. Similarity of residues for these three sequence is summarized in the fourth row (* = identical, : = strong similarity, • = weak similarity). For comparison the signal sequence of TorA is shown in the bottom row. Regions of the TorA sequence that show remarkable similarity with the consensus sequence of DmsD substrates are indicated in grey boxes. N-regions are shown in black type, H-regions in red and C-regions blue, and the two arginines of the twin-arginine motif are typed in bold.

The amino acid sequences of the signal peptides of TorA and DmsA were compared in order to assess which part(s) of the signal sequence are responsible for the specific interaction with the corresponding REMP. The signal sequences of YnfE and YnfF were included in this exercise, since these paralogs of DmsA are also known substrates of DmsD [10], [31]. First, the sequences of DmsA, YnfE and YnfF were aligned together using ClustalW (Figure 4). Apart from the consensus twin-arginine motif S/TRRxFLK that is found in all Tat substrates, the signal sequences of the DmsD substrates are remarkably similar in both the H-region and the beginning of the C-region. Second, the signal sequence of TorA was included in the alignment. Despite a similar overall composition, the signal sequence of TorA does not align so well with the other three signal sequences without the introduction of insertions and deletions (Figure 4).

**Figure 5:**
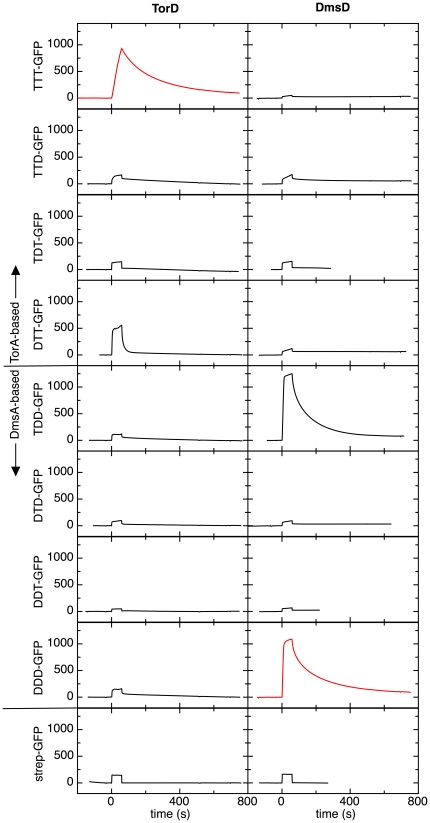
Interactions of chimeric signal sequences with REMPs. The composition of the signal sequences is shown on the left as a three-letter code. This indicates whether the N-, H-, and C-regions originate from the DmsA (D) or the TorA (T) signal sequence, respectively. For example, DTT represents a signal sequence that consists of the N-region of DmsA followed by the H- and C-regions of TorA. For each chimeric pre-protein the SPR response curve is shown for an injection of a 200 nM solution over immobilized TorD and a DmsD, respectively, as indicated at the top of the graph. The response curves for the wild-type signal sequences and their corresponding REMPs are shown in red. A response curve for 200 nM signal peptide-free Strep-GFP is shown in the bottom panel.

The high similarity of the DmsD substrates in their H- and C-regions and the lack of similarity in the same regions of the TorA signal suggest that these regions might be important for specific recognition by the correct REMP. The validity of this hypothesis was tested by creating region-swapped chimeric signal sequences, in which either the N- or the H- or the C-regions of ssTorA-GFP and ssDmsA-GFP were replaced by the corresponding region from the other signal sequence. The borders between the different regions were delineated according to the scheme shown in Figure 4. This exercise resulted in a total of eight chimera pre-protein constructs (Figure 5). These pre-proteins containing region-swapped signal peptides were injected over immobilized TorD and DmsD at different concentrations, ranging from 20 to 400 nM, of which the response curves at 200 nM are shown for all pairings in Figure 5. The main purpose of this qualitative comparison was to establish in a qualitative manner whether there is an interaction between various chimeric signal peptides and REMPs. In this experimental set, we used strep-tagged GFP lacking any signal peptide as negative control and the GFPs with wild type signal peptides (named TTT-GFP and DDD-GFP in Figure 5, respectively) as positive controls for binding.

In case of ssTorA, the only chimeric signal sequence protein that bound with reasonable affinity to TorD was the DTT-GFP fusion, in which the N-region was replaced for the one of DmsA. When either the H- or the C-region of ssTorA was replaced, binding was hardly detected even at the highest pre-protein concentration, indicating that the binding site for TorD was lost. These TorA-based chimeric pre-proteins were also tested for binding to DmsD. However, none of these TorA-based signal sequences displayed any binding to DmsD (Figure 5).

For ssDmsA, similar results were observed. Its N-region could be replaced without affecting DmsD binding, but when either the H- or the C-region was replaced, binding to DmsD could hardly be detected. Apparently, the combined H- and the C-regions but not the N-region of ssTorA and ssDmsA are essential for binding to the corresponding REMP.

### DmsD reduces membrane binding of ssDmsA-GFP

**Figure 6:**
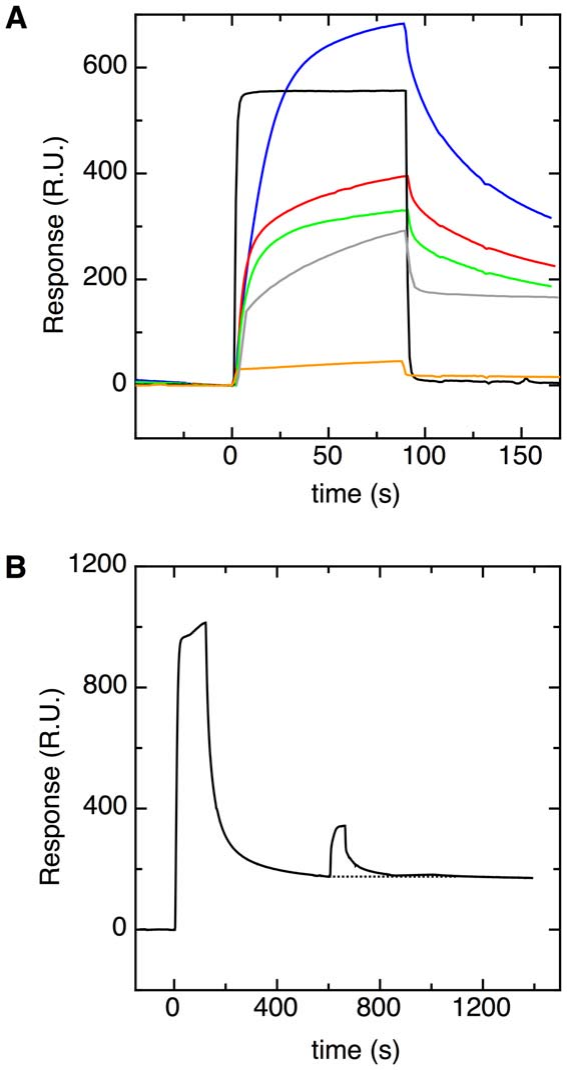
DmsD reduces membrane binding of ssDmsA-GFP. (**A**) SPR response curves of various proteins injected over a phospholipid bilayer that mimics the composition of the *E. coli* inner membrane. Injected solutions contain either 50 nM signal peptide-free Strep-GFP (orange line), 50 nM ssDmsA-GFP (blue line), 50 nM ssDmsA-GFP with 100 nM DmsD (red line), 50 nM ssDmsA-GFP with 200 nM DmsD (green line), 50 nM TorD (grey line), or 500 nM DmsD (black line). The red and green curves were corrected for the jump in refractive index by subtracting the response curves for injections of solutions containing 100 or 200 nM DmsD, respectively. (**B**) SPR response curve for an experiment in which DmsD was injected over a surface consisting of ssDmsA-GFP bound to a phospholipid bilayer. ssDmsA-GFP (100 nM) was injected over an immobilized phospholipid bilayer for a period of 100 s. The surface was washed with buffer for 500 s to remove weakly bound ssDmsA-GFP. Subsequently, buffer containing 25 nM DmsD was injected.

The second aim of this work was to find out how REMP-signal sequence binding relates to the next step in translocation. In earlier work, we showed that signal peptides mediate binding of Tat substrates to phospholipid bilayers and biological membranes, and speculated that this binding might be a step en-route to translocation [11]. Furthermore, it has been hypothesized that the role of REMP binding to signal peptides is to prevent the premature interaction of incomplete or unfolded substrates with the Tat translocase [23]. Accordingly, it would be interesting to investigate membrane binding of pre-proteins in the presence and absence of REMPs. To this end, phospholipid bilayers mimicking the *E. coli* inner membrane lipid composition were immobilized on an L1 SPR chip. First we tested whether the REMPs themselves could bind to these membrane surfaces. No interaction of DmsD alone with membranes was found (Figure 6A), which is in agreement with earlier work [16]. The step-like SPR response seen for DmsD solely reflects a change in refractive index of the injected solution. In contrast, TorD could bind to phospholipid bilayers directly (Figure 6A), and therefore the TorD-ssTorA pair was not used in this experiment. A solution containing 50 nM ssDmsA-GFP was injected over a freshly made membrane surface (Figure 6A). ssDmsA-GFP did bind to the bilayer, as reported previously [11]. Membrane binding is caused by the signal peptide, since signal peptide-free Strep-tagged GFP did not bind to the membrane (Figure 6A). Subsequently, 50 nM ssDmsA-GFP was co-injected with 100 or 200 nM of DmsD. The resulting SPR curves demonstrated that with increasing DmsD concentration, ssDmsA-GFP binding to membranes was reduced.

We then tested whether DmsD can bind to membrane-bound ssDmsA-GFP. First, a solution containing 100 nM ssDmsA-GFP was injected over the immobilized membrane. ssDmsA-GFP that was not firmly bound to the membrane was washed away by flushing with buffer for 500 s (Figure 6B). Subsequently, buffer containing 25 nM DmsD was injected over the phospholipid membrane pre-loaded with ssDmsA-GFP. DmsD bound reversibly to this surface. Since DmsD did not bind to phospholipid bilayers alone (Figure 6A), DmsD must be able to bind ssDmsA-GFP while it is associated with the bilayer. After injection of DmsD was stopped, the DmsD dissociated rapidly, and the equilibrium level of ssDmsA-GFP bound to the membrane was restored, as highlighted by the dashed line in Figure 6B. This showed that DmsD did not recruit significant amounts of ssDmsA-GFP out of the membrane.

## Discussion

The main question of this study was: how do REMPs discriminate between their cognate substrate(s) and other Tat substrates, which often share many similar features. We used an *in vitro* approach to study the interaction between REMPs and the substrate signal sequences. The key feature of this method is exposing the immobilized REMP to an intact model pre-protein in which the signal sequence of interest is fused to the N-terminus of a common cargo protein, GFP. The chimeric pre-proteins are demonstrated to be functional Tat substrates, since their expression in *E. coli* results in periplasmic accumulation of fluorescent, hence folded GFP [11], [29]. The advantage of this approach is that it closely resembles the native situation, and at the same time rules out potential specific interactions with the mature, non-signal sequence components of the substrate. Most previous studies to probe *in vitro* interactions with REMPs have employed synthetic peptides that cover various parts of the substrate signal sequences [26], [32]. However, the question is whether the structure and dynamics of such short synthetic peptides are comparable to the physiological situation, where the peptide is attached to a much larger protein cargo. Other studies have used intact pre-proteins [9], [33], which have the disadvantage that contributions to the REMP binding from the mature domain cannot be excluded. For example, TorA from *Shewanella massilia* is known to have two binding sites for TorD. One of them is in the signal sequence, while the other is in the mature domain [33]. Oresnik *et al.* previously showed that purified recombinant DmsD interacts with the pre-protein forms of DmsA as well as TorA [9]. However, here we have shown that the signal sequences of TorA and DmsA contain all the information necessary to target the cargo protein to the correct REMP. Based on the data described here, *E. coli* DmsD does not bind to the signal peptide of *E. coli* TorA. It is thus possible that DmsD can recognize a binding site in the mature domain of TorA.

The interaction between ssTorA-GFP and TorD is characterized by *K_d_* = 0.33 µM. The interaction between TorD and the signal sequence of TorA has been investigated previously by various techniques (Table 2). Most studies find *K_d_*-values around 1 µM. However, one group studied the interaction between TorD and the full-length signal peptide of TorA fused at the C-terminus of the maltose-binding protein, and found a much lower *K_d_* -value of 59 nM [32]. The influence of MBP to the affinity is unclear.

The parts of the signal sequence that are important for specific REMP binding were identified using model pre-proteins with chimeric signal sequences. The signal sequences of TorA and DmsA share little sequence identity (Figure 4), though the H-regions are at least rather similar in overall composition. Also the C-region of TorA is similar in length and polarity compared that of DmsA, and both contain a proline residue and at least one arginine. The N-region differs the most, with that of TorA being shorter and more polar. One might thus expect that the N-region, which contains the twin-arginine motif, would be important for conveying the REMP binding specificity. However, a more detailed analysis of the signal sequences shows that the H- and C-regions of DmsD substrates are rather well conserved, whereas they differ significantly in the N-region (Figure 4). This points to the combined H- and C-regions being the recognition site for DmsD. Indeed, the experiments with chimeric signal sequences (Figure 5) show that the combined H-/C-regions are essential for binding, whereas the N-region has little, if any, effect. These results are in agreement with previous work in which ITC was used to measure binding of TorD to short peptides corresponding to various parts of the TorA signal peptide [26], [32]. Interestingly, Chan *et al.* recently identified a binding pocket on the surface of *E. coli* DmsD that mainly consists of hydrophobic and aromatic residues [34]. Furthermore, our observations that the conserved arginine pair, which is essential for transport, is not crucial for REMP binding (Figures 2 and 3, Table 2) are also consistent with a central role for the H- and C-regions in REMP binding. Nevertheless, the part of the N-region in which the twin-arginine motif is located is involved in the interaction with REMPs, probably via a charge-interaction, because the mutant ssTorA(GHP)-GFP does not bind to TorD at all. The N-region is however not used to discriminate between TorA and DmsA.

Binding of the H-region to a REMP must be primarily governed by the hydrophobic effect. The entropic hydrophobic effect is driven by the exclusion of water, and on it own as such is not expected to provide specificity of the REMP towards a certain signal peptide. Some data suggest that the signal peptide binds in a particular conformation to the REMP. While being mostly unstructured in solution, signal peptides have a strong tendency to adopt an α-helical conformation in a hydrophobic membrane-mimicking environment [35], and probably also upon binding with a hydrophobic binding pocket on a REMP. Recently, Zakian *et al.* showed that the REMP NarJ binds to its cognate signal peptide ssNarG in an α-helical conformation, and that the affinity is mostly determined by the hydrophobic effect [36]. The same might be true for ssTorA binding to TorD and ssDmsA binding to DmsD.

The second question of this study was how REMP binding relates to the twin-arginine translocation mechanism. The path starts with synthesis of a twin-arginine-containing polypeptide on a ribosome in the cytoplasm, and ends with the translocation of a folded, often cofactor-containing protein to the periplasm. It was earlier shown that membrane binding of Tat pre-proteins is the last step en route to transport that precedes binding to the Tat translocon [11], [12]. Here, the possibility is assessed that REMP binding and subsequent release are the steps directly preceding membrane binding. Figure 6A shows that DmsD reduces membrane binding of ssDmsA-GFP *in vitro*. Hence, the presence of sufficient DmsD in the cytoplasm could reduce membrane binding of ssDmsA-GFP, and thereby retard transport of the pre-protein. This might be the simplest way in which REMPs perform their so-called proofreading function. If true, the twin-arginine translocation mechanism would look as follows. After synthesis of the twin-arginine-containing polypeptide on the ribosome, REMPs rapidly bind to the signal peptide of the cognate substrate with a relatively high affinity, and hence prevent premature binding of the substrate to the membrane and subsequently to the TatBC complex. While the REMP is bound to the pre-protein, other maturation enzymes can fold the protein and incorporate the appropriate cofactor. After spontaneous dissociation the REMP will usually rebind rapidly, until occasionally the released signal peptide binds to the membrane. Once membrane bound, the signal peptide is no longer fully available to the REMP, as our data show for DmsD and ssDmsA-GFP (Figure 6B). The REMP cannot recover the pre-protein out of the membrane, and the substrate will proceed to the translocation machinery. In this way, REMPs provide a time window during which the essential redox cofactors can be incorporated. The length of this time window would then be a function of the relative affinity of the signal peptide affinities for the REMP and the target membrane.

The model described above is speculative and requires further experimental investigation. Nevertheless, the model is consistent with the available information. Nucleotide hydrolysis might provide sufficient energy to release a tightly bound substrate. There is indeed some evidence that TorD binds guanosine triphosphate (GTP) [26]. However, GTPase activity was recently shown to be specific for a domain-swapped TorD homodimer [37], of which the physiological role is unclear. We do not know and cannot control the oligomeric state of TorD on the SPR chip, although the observed 1∶1 binding stoichiometry suggests that TorD is mainly monomeric on the surface (Figure 2). Nevertheless, when we measured release of ssTorA-GFP from TorD in the presence of GTP, we did not observe any effect (data not shown). We thus conclude that TorD binds ssTorA with a micromolar dissociation constant, and that there is no evidence that nucleotide hydrolysis promotes release of an intact pre-protein.

In conclusion, REMP binding to signal peptides serves at least two purposes. The first purpose, already well established, is that this interaction prevents premature cleavage of signal peptides by peptidases [19]. The second purpose is to prevent premature interactions of pre-proteins with the translocation machinery. We propose that binding of a REMP to a signal sequence delays binding of the pre-protein to the membrane and subsequently to the Tat translocase. This might provide a time frame in which the mature domain of the pre-protein can fold and the required cofactors can be incorporated.
